# MEIRLOP: improving score-based motif enrichment by incorporating sequence bias covariates

**DOI:** 10.1186/s12859-020-03739-4

**Published:** 2020-09-16

**Authors:** Nathaniel P. Delos Santos, Lorane Texari, Christopher Benner

**Affiliations:** 1 Department of Biomedical Informatics, University of California, San Diego, 9500 Gilman Drive, La Jolla, CA 92093-0640 USA; 2 Department of Medicine, University of California, San Diego, 9500 Gilman Drive, La Jolla, CA 92093-0640 USA

**Keywords:** Motif enrichment, Logistic regression, Differential analysis, ChIP-seq, MEIRLOP, Score-based

## Abstract

**Background:**

Motif enrichment analysis (MEA) identifies over-represented transcription factor binding (TF) motifs in the DNA sequence of regulatory regions, enabling researchers to infer which transcription factors can regulate transcriptional response to a stimulus, or identify sequence features found near a target protein in a ChIP-seq experiment. Score-based MEA determines motifs enriched in regions exhibiting extreme differences in regulatory activity, but existing methods do not control for biases in GC content or dinucleotide composition. This lack of control for sequence bias, such as those often found in CpG islands, can obscure the enrichment of biologically relevant motifs.

**Results:**

We developed Motif Enrichment In Ranked Lists of Peaks (MEIRLOP), a novel MEA method that determines enrichment of TF binding motifs in a list of scored regulatory regions, while controlling for sequence bias. In this study, we compare MEIRLOP against other MEA methods in identifying binding motifs found enriched in differentially active regulatory regions after interferon-beta stimulus, finding that using logistic regression and covariates improves the ability to call enrichment of ISGF3 binding motifs from differential acetylation ChIP-seq data compared to other methods. Our method achieves similar or better performance compared to other methods when quantifying the enrichment of TF binding motifs from ENCODE TF ChIP-seq datasets. We also demonstrate how MEIRLOP is broadly applicable to the analysis of numerous types of NGS assays and experimental designs.

**Conclusions:**

Our results demonstrate the importance of controlling for sequence bias when accurately identifying enriched DNA sequence motifs using score-based MEA. MEIRLOP is available for download from https://github.com/npdeloss/meirlop under the MIT license.

## Background

Transcription factors (TFs) mediate transcriptional responses, inducing or repressing transcription of genes by binding to DNA at regulatory regions and recruiting RNA polymerase or accessory factors [1]. Motif enrichment analysis (MEA) of regulatory sequences is a method used to identify over-represented DNA sequence patterns (motifs) in regulatory regions [2, 3]. Bioinformatics methods usually represent TF binding motifs as position weight matrices (PWMs), which describe the DNA-binding specificities of transcription factors, and can predict potential binding sites in regulatory regions [4, 5]. Researchers use the enrichment of TF binding motifs to infer which TFs may contribute to specific transcriptional responses by binding those motifs in regulatory region sequences [6]. This enables the inference of which TFs mediate a cell’s transcriptional response to a condition, such as infection or disease state, marking those TFs as potential therapeutic targets.

**Fig. 1:**
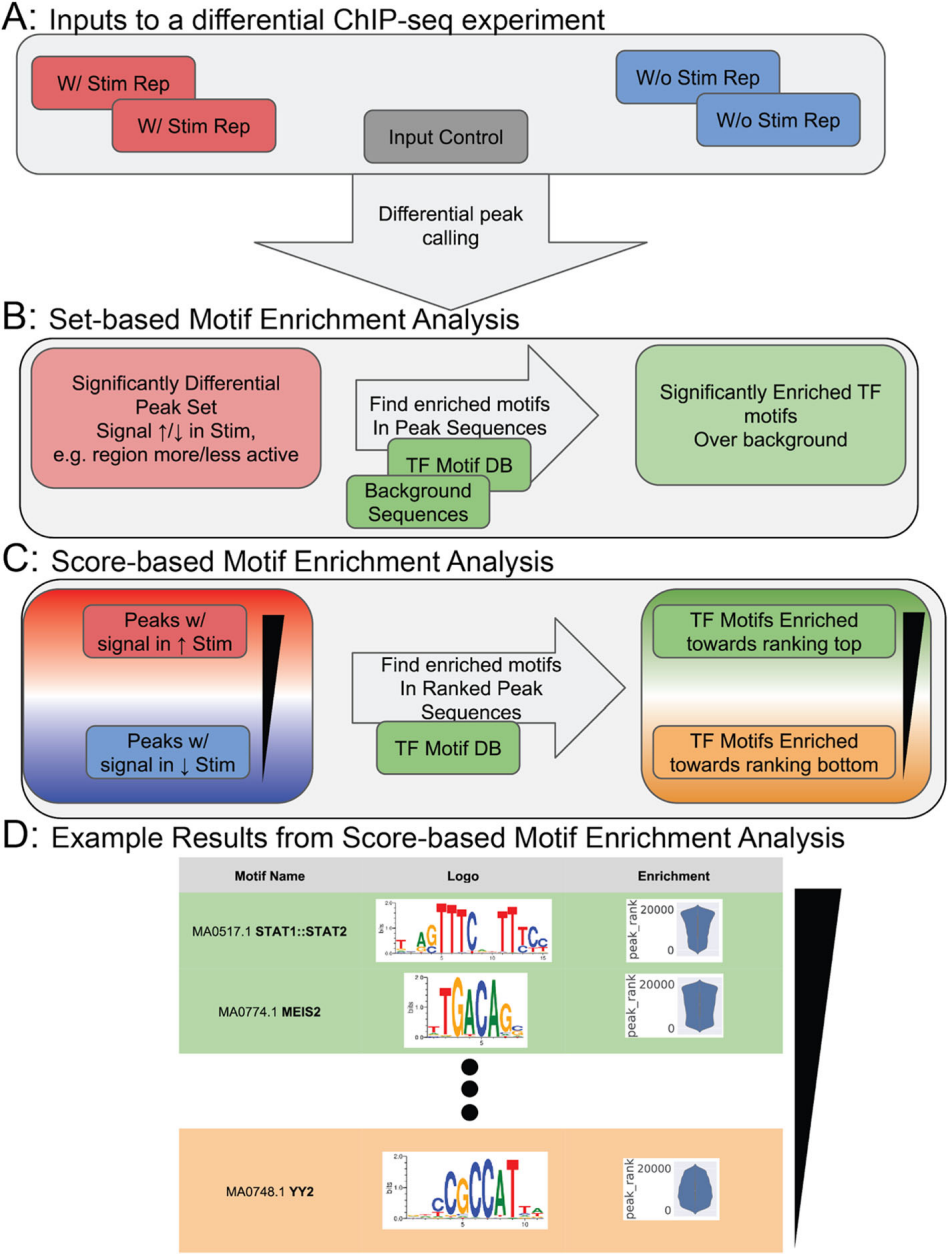
Motif enrichment analysis can be based on discrete sets of sequences or scored sequences. **a** Diagram of inputs to a typical differential ChIP-seq binding experiment, consisting of replicates in stimulated and unstimulated conditions, and an input experiment(s). **b** Diagram of set-based motif enrichment methods. These methods receive significantly differentially regulated subsets of regulatory sequences (e.g. peaks) and return a significantly enriched subset of TF motifs. **c** Diagram of score-based motif enrichment methods. These receive a list of scored sequences based on differential signal between stimulated and unstimulated conditions, then return TF motifs enriched towards the top or bottom of the scored sequences. **d** Illustration of the potential output of a score-based enrichment method, featuring motif name, motif logo, and a description of the enrichment of a motif towards the top or bottom of scored sequences

When using most MEA methods, regulatory regions are typically scored by their biological response in transcriptomic or epigenomic assays. For example, H3K27ac localization on chromatin serves as a marker for active regulatory regions [7]. MEA on differential H3K27ac ChIP-seq data can reveal which motifs and TFs regulate transcription in regions that change their activity in response to stimulation [8]. Researchers typically filter these regulatory regions by a score threshold and place them into sets to yield contrasting categories (e.g., regulatory regions with higher activation levels after stimulation vs. those with lower activation levels after stimulation) (Fig. 1a,b). Then, motif scanners detect motifs in sequences within those categories, followed by set enrichment tests (e.g. the Fisher exact test) which determine the overrepresentation of motifs in each category. This allows the imputation of motifs and transcription factors that influence transcriptional response in those conditions (Fig. 1b). We term this process set-based MEA, for its thresholding of sequences into categorical sets.

However, this thresholding ignores how the magnitude of a regulatory region’s response can occur along a continuum, and removes relevant information from the score: For example, by collapsing strongly activated and weakly activated regulatory regions into the same set of “activated regulatory regions”, set-based MEA obscures potentially important differences between those two categories. In addition, preset thresholds can lead to few regulatory regions being present in one or more sets, reducing confidence in downstream enrichment results. Such sparsity can occur because of technical reasons: the choice of underlying normalization and differential assay analysis influences the number of genomic features that pass thresholds for differential expression/activation [9, 10]. When this occurs, a researcher may attempt to revisit the underlying scores, applying different thresholds to achieve a suitable enrichment: However, after testing many thresholds, a significant enrichment can appear purely by chance. This motivates the use of score-based MEA that uses the biological response scores of regulatory regions directly.

Threshold-free motif enrichment asks: Given a list of TF binding motifs and a sorted list of scored/ranked sequences, what motifs are enriched at the top or bottom of that list (Fig. 1c, d)? This follows from the fact that most biological data, differential or otherwise, naturally lends itself to ranking [11]. While individual regulatory regions may not fulfill pre-set thresholding criteria for significantly differential signal, motifs may appear in regulatory regions biased towards the top or bottom of a list, showing a correlation with the quantities underlying any potential differential signal thresholds. This concept has precedence in gene expression and ontology analyses: GSEA Prerank identifies significantly enriched gene sets at the top or bottom of a differential ranking of genes [12].

Related work has developed multiple score-based MEA methods for inferring relevant TFs. A handful of implementations share many similarities with set-based MEA, except that they determine a data-driven partitioning threshold along the scored regulatory regions. The chosen threshold maximizes the resulting enrichment from a Fisher exact test or hypergeometric test [13, 14]. An alternative method is to perform a rank-sum test, comparing the score ranks of regulatory regions that contain a motif against those that do not. Other strategies involve finding the correlation between the strength of a motif in a regulatory region’s sequence against the score of the regulatory region, under the reasoning that stronger instances of a relevant motif should contribute more to the score. The software package AME (from the MEME suite) implements many of these strategies, including variable threshold Fisher exact tests, rank-sum MEA, and correlation with Pearson coefficients for linear correlation, as well as Spearman coefficients for rank correlation [2].

One complication that can arise when performing MEA is that a nonrandom distribution of nucleotide or dinucleotide frequencies can lead to a bias in sequence content among sets of regulatory region sequences. Thus, MEA methods that do not control for this effect may assign significance to a motif that preferentially recognizes sequences favored by the biased sequence composition. For example, CpG islands are present in promoter regions of genes, so a motif scanner is more likely to observe GC-rich motifs there by chance. Similarly, dinucleotides found in the first two bases of codons may be over-represented in DNA sequence sampled near coding exons, and thus can contribute to spurious enrichment values for motifs over-represented near intron borders [15]. Multiple set-based MEA methods control for this effect: AME and CentriMo from the MEME Suite allow specification of a control sequence file for comparative motif enrichment, and can generate the control/background sequences by shuffling dinucleotides from the target sequence set [2, 3].. HOMER can automatically select sequences from a reference genome that resemble the GC content of a target set of motifs, and can also re-weight a sequence’s contribution to a contingency table to minimize differences in weighted dinucleotide frequency between sets [16]. Methods such as Pscan/Pscan-ChIP, CLOVER, BiasAway, and GENRE generate or retrieve background sequences to control for sequence properties of a target sequence set [6, 15, 17–19]. However, such control is absent in current score-based MEA methods that identify motifs through a PWM.

To provide a score-based MEA method more robust to covariate effects, we developed MEIRLOP (Motif Enrichment In Ranked Lists of Peaks) [20]. MEIRLOP uses logistic regression to model the probability of a regulatory region sequence containing a motif as a function of a regulatory region’s activity score. To account for sequence bias, our method derives covariates from the dinucleotide frequencies of the regulatory region sequences, and incorporates these into the logistic regression model. The regression model’s coefficient for the score then summarizes whether a motif is more likely to appear in regulatory regions with higher or lower response scores. In addition, we avoid multicollinearity in the logistic regression model by reducing the covariates into a set of principal components summarizing 99% of the variance. This dimensional reduction maintains the stability of the regression model and improves estimation of regression coefficients [21].

Although prior works have used logistic regression in motif analysis, these methods have not used quantitative changes in regulation as the predictor variable. Keles et al. employ logistic regression as another form of set-based MEA: They use the presence or absence of a motif as a predictor of whether a sequence is in a target set [22]. Yao et al. set binding site presence or absence as outcome variables of logistic regression. However, their method uses a predictive variable derived not from sequencing coverage of regulatory regions, but from the overall count of motifs in a region’s sequence [23].

MEIRLOP enables enrichment of biologically relevant TF binding motifs where other methods may fixate on nucleotide or dinucleotide sequence bias. On differential H3K27ac data, it achieves superior accuracy over other methods in identifying significant enrichment of motifs known to mediate interferon signaling response. On ENCODE ChIP-seq data, it achieves improved performance relative to other score-based MEA methods implemented in AME, but returns results over twenty times faster. Finally, we demonstrate how MEIRLOP can be applied to a variety of different NGS profiling methods and different activity scores to improve the identification and interpretation of functionally relevant TF motifs.

## Implementation

### MEIRLOP motif enrichment procedure

MEIRLOP is based on a logistic regression model for motif enrichment. At minimum, it accepts a list of scored sequences (in the AME scored FASTA format, with sequence headers consisting of a name and score separated by a space), and a motif database (in JASPAR format). MEIRLOP’s novel motif enrichment procedure is executed in three parts, described below:

1. Scanning sequences for motifs
2. Principal component reduction of covariates
3. Logistic regression for motif enrichment

#### Scanning sequences for motifs

To detect transcription factor binding motifs in genomic sequence, we use the MOODS motif scanner to scan for sequence matching PWMs from the input motif set, which is provided to MEIRLOP in the JASPAR format [4, 24]. In our command lines, we refer to this motif set with the filename: “jaspar.txt”.

The MOODS motif scanner internally takes two parameters to determine if a subsequence matches a motif matrix: a pseudocount and a *p*-value, which default to 0.001. MEIRLOP sets these parameters using arguments ‘--pcount’ and ‘--pval’.

In this work we use the JASPAR 2018 CORE vertebrate non-redundant motif set, which consists of 579 motifs [4]. We selected this motif set to limit redundancy between different versions of what are essentially the same motif (i.e. avoid many motifs matching the same transcription factor), and to restrict the motifs tested to those relevant for analyzing human data. In addition, this set of motifs is available in formats compatible with AME, facilitating comparison of our method with AME.

#### Principal component reduction of covariates

Although it is possible to directly input sequence-derived covariates into a logistic regression model, when using the k-mer frequencies of sequences as covariates, the multicollinearity of these frequencies (due to e.g. CpG islands) can lead to model instability and inaccurate parameter estimation [21]. To account for this while preserving the ability to control for k-mer frequencies, we adapt the strategy of Aguilera et al.: We reduce multiple k-mer frequency covariates into a lower-dimensional set of principal components [21], converting the multicollinear predictors into a set of linearly uncorrelated predictors explaining 99% of the variance. We use the PCA implementation available in scikit-learn [25]. We refer to a single resulting reduced covariate as *x*_*c*_.

By default, MEIRLOP controls for dinucleotide frequency covariates, but this behavior can be adjusted with the ‘--kmer’ argument to control for single nucleotide frequencies (‘--kmer 1’) or no covariates (‘--kmer 0’). Additional experimentally derived covariates can be incorporated using the ‘--covariates’ argument.

#### Logistic regression for motif enrichment

Although prior work has previously applied logistic regression to the analysis of transcription factor binding sites [22, 23], we present a different logistic regression model that more closely follows the example of linear regression as applied in AME [2], while controlling for the effect of collinear covariates (e.g. short k-mer frequencies).

Let *p* be the probability of a sequence containing a given motif *m*, and let *x*_*s*_ be the score assigned to the sequence, e.g. H3K27ac ChIP-seq signal. We then model the log-odds of a sequence with score *x*_*s*_ containing motif *m* as:

$$ \mathit{\log}\left(\frac{p}{1-p}\right)={\beta}_0+{\beta}_s{x}_s+\sum \limits_c^n{\beta}_c{x}_c $$

Where *x*_*c*_ refers to one of the *n* reduced covariates previously described, with *β*_*c*_ being the corresponding coefficient. After maximum likelihood estimation of the coefficients and bias term, *β*_*s*_ can be interpreted as the change in the log-odds of a sequence containing motif *m*, for a one unit increase in *x*_*s*_. To ensure that the maximum likelihood estimation converges, all predictor variables are standardized. The significance of the coefficient *β*_*s*_ is determined using the Wald test, as per the Statsmodels implementation of logistic regression [26]. To control for multiple hypothesis testing across multiple motifs, we applied Benjamini-Hochberg correction to the Wald test *p*-values [27].

This approach to motif enrichment allows controlling for sequence derived covariates such as GC content and k-mer frequencies, allowing the model to compensate for sequence bias similarly to certain set-based methods such as HOMER [16].

MEIRLOP displays logistic regression results using an interactive HTML table powered by Datatables.net, with motif logos generated by Logomaker [28].

### Differential analysis of IFN-β stimulation against control

We started with cell culturing and ChIP-seq for data acquisition. This was followed by differential ChIP-seq bioinformatics analysis comparing stimulated samples vs. unstimulated controls. Finally, we ran motif enrichment analysis on the differential ChIP-seq peaks using MEIRLOP and AME in different configurations.

We conducted the data acquisition in two parts, described below:

1. Cell culture and treatment
2. ChIP-seq and Crosslinks
  The data analysis and comparison was conducted in three parts, also described below:
3. Differential ChIP-seq analysis (preprocessing)
4. Motif enrichment analysis (running MEIRLOP & AME)
5. Motif enrichment accuracy evaluation

#### Cell culture and treatment

HCT116 CMV-osTIR1 RAD21-mAC cells were obtained from Masato T. Kanemaki [29] and cultured in McCoy’s 5A medium supplemented with 10% FBS. Cells were grown in a 37 °C incubator with 5% CO2. Cells were treated with 0.1% final DMSO for 6 h and then treated with either IFN-β (1000 unit/ml) for one hour or not further treated.

#### ChIP-seq and crosslinking

Crosslinking and ChIP-seq was performed as described in Heinz et al., 2018 with few adjustments [30]. Briefly, cells were fixed directly by adding formaldehyde into media to a final concentration of 1% formaldehyde for 10 min at room temperature and quenched with 125 mM Glycine. Cells were then pelleted at 300 g for 5 min at 4 °C, washed twice with cold PBS (with 0.5% BSA), snap frozen in liquid nitrogen and stored at − 80 °C.

ChIP-seq was performed on 500,000 cells as described in Heinz et al., 2018 [30]. H3K27ac antibodies were obtained from Active Motif (cat#:39133). Libraries were single-end sequenced for 84 bp to a depth of 5.5–8.6 reads on an Illumina NextSeq500 instrument.

#### Differential ChIP-seq analysis

After sequencing and adapter trimming with FastP [31], 5.5 M - 8.6 M reads per library were aligned to the GRCh38 reference genome using bowtie2 at overall alignment rates of 98.5–99.5% [32]. For each stimulation (*n* = 2) and control sample (*n* = 2), MACS2 was used to call peaks of median length 1kbp relative to a background input sample (of 16 M reads) [33]. DiffBind was used to call differentially acetylated peaks between stimulated and unstimulated conditions [34]: Internally, DiffBind counts reads from each sample within each peak, then uses DESeq2 to calculate differential statistics such as log 2 fold change while accounting for library size and replicate/batch effects.

In order to obtain scores for these peaks and their sequences, we used the log2 fold change for each feature as called by DiffBind as the score. For each peak, the sequence +/− 500 bp of each feature’s center was extracted. The motif enrichment analysis methods we evaluated then received these scores and sequences for input, in the scored FASTA file “deseq2-ifnb.diff-peaks.scored.fa”.

#### Motif enrichment analysis

When evaluating MEIRLOP with dinucleotide frequency covariates, we used the command:

“OMP_NUM_THREADS=25 $(which time) --verbose python -m meirlop --jobs 25 **--kmer 2** --pcount 0.01 --pval 0.001 --html --scan --fa deseq2-ifnb.diff-peaks.scored.fa jaspar.txt {meirlop_output_directory}”.

The argument “--kmer **2**” sets MEIRLOP to use **di**nucleotide frequency covariates. The file “deseq2-ifnb.diff-peaks.scored.fa” is available in the “data” directory of the MEIRLOP Github repository.

When evaluating MEIRLOP without covariates, we used the command:

“OMP_NUM_THREADS=25 $(which time) --verbose python -m meirlop --jobs 25 **--kmer 0** --pcount 0.01 --pval 0.001 --html --scan --fa deseq2-ifnb.diff-peaks.scored.fa jaspar.txt {meirlop_output_directory}”.

The argument “--kmer **0**” sets MEIRLOP to use **no** k-mer frequency covariates.

MEIRLOP gives positive enrichment values for motifs overrepresented in sequences with higher scores, while AME gives positive enrichment values for motifs overrepresented in sequences with lower scores. Due to this difference in scoring/enrichment conventions we must transform the input sequence scores for AME relative to those used by MEIRLOP: we multiplied scores by -1 prior to input for AME and its implemented methods [2]. AME version 5.0.2 was used unless otherwise specified [2].

#### Motif enrichment accuracy evaluation

To assess motif enrichment results across multiple methods, we adopt the strategy previously used to assess different motif enrichment methods in AME [2]. We reduce motif enrichment scores to the percentile rank of the motif enrichment score. The formula for this percentile rank accuracy (PRA) metric is reproduced below from eq. 8 of McLeay & Bailey 2010 [2]:

$$ PRA=\frac{R_k}{N} $$

Where *R*_*k*_ is the rank of the target motif *M*_*k*_ within a method’s enrichment results, and *N* is the total number of motifs in the database. Since true positive results are expected to have larger positive enrichment scores in these method comparison experiments, *R*_*k*_ is higher for more highly positive scores and reflects a better result for a method.

Exclusion from the results table of any method defaults to a rank score of 0, and ties resolve to the lowest applicable rank. This penalizes methods that do not provide results for the correct motif, or that simply assign many motifs the same high enrichment score. For methods implemented in AME, the rank is higher the earlier the motif appears in AME’s result table. For our method, the rank is correlated with the logistic regression coefficient of regulatory region score against motif presence. To obtain percentile ranks, the rank is divided by the number of motifs in the reference motif database.

We use the percentile rank accuracy metric to assess the accuracy of both AME and MEIRLOP, so this calculation is not implemented in MEIRLOP itself, but is instead evaluated after running both AME and MEIRLOP.

### Comparison of MEIRLOP and AME on ENCODE ChIP-seq data

We started by selecting ENCODE ChIP-seq experiments and extracting scored FASTA files for input into MEIRLOP and AME. Then, we ran MEIRLOP and AME to analyze motif enrichment in the scored sequences in these FASTA files and evaluated each method’s performance using the percentile rank accuracy metric previously described.

This comparison was conducted in three parts, two of which are described below:

1. ENCODE ChIP-seq experiment selection and sequence extraction (preprocessing)
2. Motif enrichment analysis on ENCODE ChIP-seq data (running MEIRLOP & AME)
3. Motif enrichment accuracy evaluation (as previously described)

#### ENCODE ChIP-seq experiment selection and sequence extraction

In order to determine the performance of logistic regression with covariates on other ChIP-seq datasets, we used MEIRLOP on scored ChIP-seq peaks from 582 ENCODE ChIP-seq TF binding experiments, summarized in supplementary Table S3 [35, 36]. These were assays on human cell lines for which the ChIPed TF could be matched to a TF binding motif in the JASPAR 2018 non-redundant motif database by gene name. These experiments were selected to exclude those with severe (red flag) audit categories. From each experiment, approximately 300K peaks were obtained, with peak scores assigned by SPP. Peaks were obtained prior to filtering for IDR in order to obtain enrichments across a range of peaks including those lacking high affinity binding motifs. Motif enrichment was restricted to sequence within +/− 200 bp of the peak center.

#### Motif enrichment analysis on ENCODE ChIP-seq data

MEIRLOP was run with covariates controlling for sequence dinucleotide frequencies. MEIRLOP was invoked multiple times on data for different ENCODE accession numbers. E.g., for ENCODE accession ID “ENCSR976TBC”, MEIRLOP was run using the command line:

“OMP_NUM_THREADS=25 $(which time) --verbose python -m meirlop --jobs 25 --html --pcount 0.1 --pval 0.001 --kmer 2 --fa scored_fastas/ENCSR976TBC.fa jaspar.txt meirlop_outputs/ENCSR976TBC”.

We parallelized AME runs using GNU Parallel [37]. As described previously, sequence scores were inverted for use with AME.

### Differential csRNA-seq analysis

We started by extracting sequences and scores for differential TSS data previously generated as described in Duttke et al. [38]. We then ran MEIRLOP on the scored TSS sequences.

This analysis was performed in two parts, described below:

1. TSS sequence extraction and scoring (preprocessing)
2. MEIRLOP analysis of TSS

#### TSS sequence extraction and scoring

Differential TSS were found from murine BMDMs as described in Duttke et al. [38]. We performed enrichment for motifs found within +/− 150 bp of the TSS, and scored sequences by the differential log 2 fold change between KLA stimulated and unstimulated control conditions as computed by HOMER getDiffExpression.pl (which wraps DESeq2 to calculate differential statistics while accounting for library size and replicates).

#### MEIRLOP analysis of TSS

MEIRLOP was run with covariates controlling for sequencing dinucleotide frequencies. We ran MEIRLOP with the command line:

“OMP_NUM_THREADS=30 $(which time) --verbose python -m meirlop --jobs 30 --kmer 2 --pcount 0.001 --pval 0.001 --html --scan --sortabs --bed differential_tss.bed --fi genome.fa jaspar.txt differential_tss.meirlop”.

Using MEIRLOP with input bed files this way requires bedtools version 2.29.0 [39, 40]: Later versions (specifically 2.29.2) have incompatible behavior for the “bedtools getfasta” subcommand. The file “differential_tss.bed” is available in the “data” directory of the MEIRLOP Github repository.

### Analysis of DHS scored by histone ChIP-seq ratios

We started by retrieving DNase-seq and histone ChIP-seq data from ENCODE, then extracting sequence, coverage, and signal data [35, 36]. These were converted into scored sequence FASTA files and a covariate file, where each entry in these files corresponded to one DHS. We then ran MEIRLOP on the scored DHS sequences and their covariates.

This analysis was performed in two parts, described below:

1. DHS sequence extraction
2. MEIRLOP analysis of DHS

#### DHS sequence extraction, scoring, and covariates

DHS for K562 cells were taken from ENCODE DNase-seq experiment accession ENCSR000EOT [35, 36]. We used the narrowPeak BED file ENCFF821KDJ for the BED intervals [35, 36]. DHS were then resized to +/− 75 bp around the DNase-seq peak center. To score DHS by DNase-coverage scores as a covariate, we converted the ENCODE DNase-seq bam file ENCFF156LGK into a coverage bigwig using deeptools bamCoverage, then ran deeptools multiBigwigSummary to obtain the mean values across the DHS [35, 36, 41]. To score DHS by histone-ChIP-seq, we downloaded alignments for H3K27ac, H3K27me3, H3K4me3, and H3K4me1 ChIP-seq from ENCODE, corresponding to ENCODE biosamples ENCBS639AAA (isogenic replicate 1) and ENCBS674MPN (isogenic replicate 2) [35, 36]. Details on these downloaded files are available in supplementary Table S4. We obtained obtained bigwigs corresponding to log_2_ ratios of histone ChIP-seq coverage (H3K27ac over H3K27me3, and H3K4me3 over H3Kme1) for each replicate using bamCompare [41]. Then, we ran bigwigCompare to obtain a single bigwig for each log_2_ ratio summarizing the mean across both replicates. Finally, multiBigwigSummary was used to obtain the mean values for these log_2_ ratios across genomic ranges +/− 500 bp of the center for each DHS. DHS were assigned scores from the mean histone log_2_ ratios corresponding to their centers.

#### MEIRLOP analysis of DHS

MEIRLOP was run to analyze these scored DHS sequences with covariates controlling for dinucleotide frequencies and DNase-seq coverage for each DHS. MEIRLOP was invoked multiple times for each histone ratio. E.g., for sequences scored by log2 ratio of **H3K27ac over H3K27me3**, while controlling for dinucleotide frequencies and **DNase-seq signal as a covariate** we used the command line:

“OMP_NUM_THREADS=45 $(which time) --verbose python -m meirlop --jobs 45 **--kmer 2 --covariates dhs.dnase.covariates.tsv** --pcount 0.001 --pval 0.001 --html --scan --sortabs **--fa dhs.h3k27ac_over_h3k27me3_mean.scored.fa** jaspar.txt {h3k27ac_over_h3k27me3_meirlop_output_directory}”.

A “walkthrough” Jupyter notebook for this analysis, starting from ENCODE downloads, is available on the MEIRLOP Github repository. To perform the analysis without the custom DNase-seq signal covariate, we ran a similar command but without the “--covariates” argument.

## Results

### MEIRLOP uses covariates to accurately call enrichment of relevant TF motifs

To show the utility of our method, we performed a differential motif enrichment analysis of regulatory elements modulated by interferon beta (IFN-β) treatment in HCT116 cells as measured by H3K27ac ChIP-seq. IFN-β treatment stimulates the type I interferon pathway, which ultimately leads to the activation of STAT and IRF family transcription factors to stimulate the expression of genes with antiviral activity [42]. While the type I interferon pathway is responsive in most cell types, HCT116 and other cell lines often exhibit a limited response to IFN-β treatment relative to primary myeloid cells [30], making their analysis more challenging. MACS2 found initial H3K27ac peaksets in each H3K27ac replicate and condition relative to input; then diffBind found 20,087 total H3K27ac peaks of median length 1kbp in HCT116 cells after controlling for coverage in an input experiment, and merging adjacent peaks across IFN-β- and mock-treated (control) conditions. Using diffBind’s DESeq2 analysis to find differentially acetylated peaks, we found only 2 peaks with significantly increased H3K27ac signal after stimulation and accounting for library size and replicates (adjusted *p*-value < 0.05, log 2 fold-change > 1.0) (Figure S1). As a result, set-based MEA on this small set of 2 peaks failed to return results for two known IFN-β response relevant motifs.

**Fig. 2:**
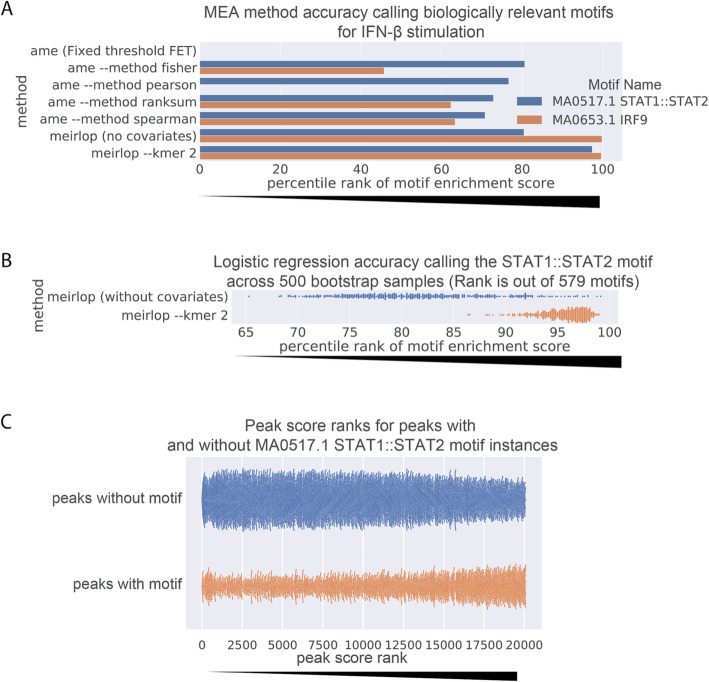
Logistic regression with covariates recovers motifs mediating IFN-β stimulation response in differentially acetylated ChIP-seq peaks**. a** Ranks of the motif enrichment scores for motifs bound by TFs activated by IFN-β across score-based MEA methods. Higher percentile ranks indicate the motif considered more significantly enriched relative to other motifs in the results of each method. Motifs excluded from enrichment reports are assigned a rank of 0. **b** Reproducibility of logistic regression accuracy of MEIRLOP when calling enrichment of the STAT1::STAT2 binding motif, with and without controlling for dinucleotide frequency covariates. Each point represents enrichment results on a bootstrap sample of the same dataset. **c** Swarm plot depicting the distributions of score ranks for peaks with and without STAT1::STAT2 binding motif instances. Each point represents a scored peak

MEIRLOP uses logistic regression and does not rely on thresholding of regulatory region sequences into sets. We extracted the central 1kbp sequence of each peak and scored the response of each regulatory region by the log_2_ fold-change of the H3K27ac signal with and without IFN-β stimulation. PCA yielded 11 covariates summarizing 99% of the variance in dinucleotide frequencies across these sequences. When accounting for PCA-based covariates derived from dinucleotide frequency, our method can identify significant enrichment of known relevant motifs in the top 10 results: These are the motifs for IRF9 and the STAT1::STAT2 heterodimer, which together bind to interferon-sensitive response elements to activate pathways in the innate immune response [42]. Upon ranking the enrichment scores of these two relevant binding motifs compared to 577 others present in the motif database, we find that the IRF9 motif achieves a percentile ranking of 99.6 (rank 577 out of 579, just below similar IRF motifs), while the STAT1::STAT2 motif achieves a percentile ranking of 97.4 (rank 564 out of 579) (Fig. 2a). Thus, our method can call enrichments of relevant TF binding motifs in differential ChIP-seq analysis, even when the difference between conditions is subtle and difficult to quantify with conventional approaches such as diffBind [43].

To establish the effect of sequence bias on the analysis, we repeated the enrichment without controlling for covariates derived from dinucleotide frequencies. While it slightly improved the enrichment for the IRF9 motif (percentile rank 99.8; rank 578 out of 579), the STAT1::STAT2 motif no longer appeared in the top ten enrichment results (percentile rank 80.5; rank 466 out of 579) (Fig. 2a, Figure S2A). Instead, more AT-rich homeobox transcription factors appeared ahead of the STAT1::STAT2 motif, comprising most of the top results (Figure S2A).

To determine whether the covariate-dependent difference in enrichment of the STAT1::STAT2 motif was because of chance, we created 500 bootstrap samples of 20,087 scored regulatory region sequences by sampling the original scored sequences with replacement, and performed motif enrichment on these with and without covariates. We found that the change in the rank of the STAT1::STAT2 motif enrichment was significant (Wilcoxon *p*-value 1.33e-83), with an average rank of 553.7 with covariates, and 471.5 without (Fig. 2b). When performing a limited enrichment controlling only for GC content, we found that GC content negatively correlated with the probability of a regulatory region sequence containing the motif (logistic regression coefficient − 0.90, Wald test p-value < 0.01), consistent with the GC-poor composition of the motifs that appear ahead of the STAT1::STAT2 motif in the enrichment results. Despite the effect of GC composition, STAT1::STAT2 appears more frequently in the sequences of higher ranked peaks than in lower ranked peaks (Fig. 2c). Thus, controlling for sequence bias allows our logistic regression-based method to accurately call the enrichment of TF motifs relevant to IFN-β stimulation.

### MEIRLOP outperforms other score-based MEA in differential ChIP-seq analysis

To evaluate the performance of our method relative to multiple approaches to score-based MEA, we applied multiple methods implemented in AME to the analysis of the 20,087 regulatory region sequences scored for IFN-β response. We found that most of the top enriched motifs found by these methods were homeobox transcription factors, consistent with the results of MEIRLOP when not controlling for sequence bias (Fig. 2a, S2A,B). IRF9 and STAT1::STAT2 motifs never obtained enrichment scores past percentile rank 86.3 (rank 500 out of 579) (Fig. S2C). Thus, multiple score-based motif enrichment methods that do not control for sequence bias cannot accurately call the enrichment of relevant TFs in our differential ChIP-seq analysis in their top results.

### MEIRLOP achieves similar or better accuracy on TF ChIP-seq data

To determine the relative accuracy of our method compared to more commonly used MEA implementations across a wide range of experiments, we analyzed scored genomic regions from a set of 23 ENCODE TF ChIP-seq experiments on H1 human embryonic stem cells (H1-hESCs) [35, 36] that analyzed TFs with known motifs in the JASPAR 2018 motif database [4]. From these scored regions, we extracted 200 bp sequences from the center of the peak for motif enrichment testing, yielding approximately 300,000 scored sequences per experiment. To evaluate the accuracy of similarly implemented MEA methods, we compared our method’s accuracy against the accuracy of AME’s implementations on the same datasets of scored regions. Similar to the differential ChIP-seq analysis, the evaluation metric was the rank of the enrichment score of the known motif for the ChIPed TF.

**Fig. 3:**
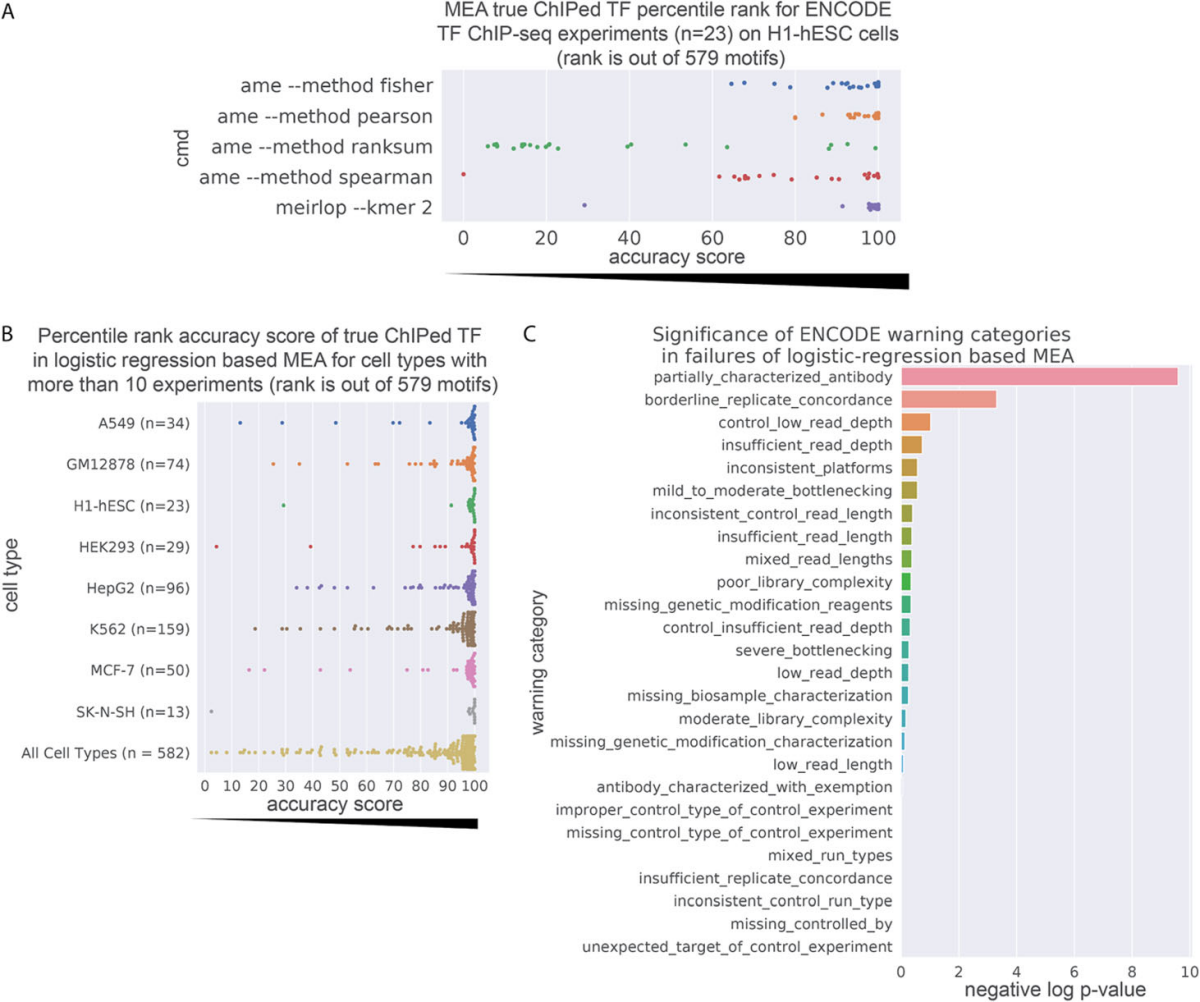
Logistic regression with covariates accurately calls motifs for ChIPed TFs in ENCODE experiments. **a** Plot of the rank for the enrichment score of the ChIPed TF’s motif in 23 ENCODE H1-hESC ChIP-seq experiments, across multiple score-based MEA. **b** Plot of the rank for the enrichment score of the ChIPed TF’s motif across multiple ENCODE cell lines. Only cell lines with more than 10 experiments analyzed are represented, as well as a separate category summarizing all cell lines. **c** Bar plot of Fisher exact test significance for enrichment of ENCODE warning flags among 36 (out of 582 eligible) experiments for which logistic regression with covariates failed to call the true ChIP-ed TF as significant, showing partial characterization of ChIP-seq antibodies as a significantly enriched warning term (Fisher exact test *p*-value = 6.8e-5, FDR = 1.563e-3)

Our method (meirlop --kmer 2) achieved an average percentile rank of 95.9 (rank 555.2 out of 579) when recovering the motif corresponding to the true ChIPed TF (Fig. 3a). Scored MEA using Pearson correlation coefficients achieved an average percentile rank of 95.4 (rank 552.5 out of 579), not significantly different from our method’s performance (Wilcoxon *p*-value = 0.09). Other MEA methods achieved lower average ranks (Fig. 3a). Thus, logistic regression can achieve accuracy similar to or better than existing score-based MEA methods on scored sequence data from TF ChIP-seq experiments. However, MEIRLOP is multithreaded and takes less time to achieve similar accuracy compared to other methods, taking an average of 10.8 min per experiment, while the Pearson correlation method in AME took 21.9 h on average.

To determine the performance of our method on TF ChIP-seq for other cell types, we used MEIRLOP to measure the enrichment of motifs in scored ChIP-seq peaks from 582 ENCODE ChIP-seq TF binding experiments on human cell lines. On average, our method achieved a percentile rank of 93.3 (rank 540.1 out of 579), placing the motif for the ChIPed TF towards the top 90% of enrichment results (Fig. 3b).

Our method failed to call significant enrichment of a motif for the true ChIPed TF in 36 of 582 experiments. To diagnose these failure cases, we retrieved ENCODE audit warning flags for each of the 582 experiments. From these warning flags, 26 of the 582 experiments had the “partially characterized antibodies” warning. Of the 36 experiments that our method failed, 8 had this warning flag present and enriched (Fisher exact test *p*-value = 6.8e-5), with the enrichment remaining significant after multiple testing of 22 other warning flags (adjusted p-value = 1.767e-3) (Fig. 3c). Thus, the antibodies used in these ChIP-seq experiments may not have been specifically targeting the proper TFs, leading to a failure to identify the expected known motif.

### MEIRLOP identifies motifs of TFs mediating KLA response from csRNA-seq

**Fig. 4:**
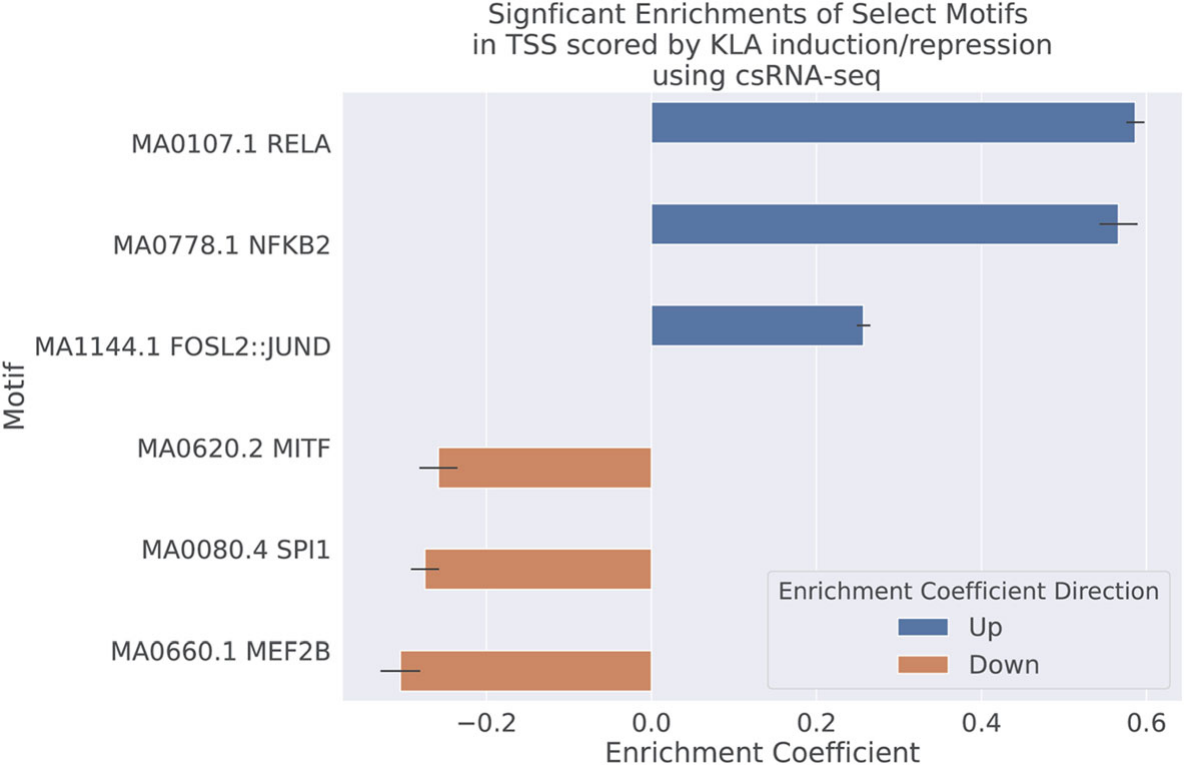
Logistic regression with covariates finds motifs for TFs mediating KLA response. Bar plot of significant logistic regression coefficients for select motifs on csRNA-seq TSS scored by differential nascent transcription signal between KLA stimulated and control conditions

Score-based MEA is well suited to analyze changes in regulatory states by incorporating the magnitude of transcriptional change into the motif enrichment calculation. To demonstrate our method’s efficacy when analyzing changes in transcription with diverse data types, we applied MEIRLOP to the analysis of capped short (cs)RNA-seq data generated in macrophages activated by TLR4-agonist KLA for 1 h or mock-treated controls. csRNA-seq is an approach that isolates initiating transcripts at both promoters and enhancers to directly assess the transcriptional activity of regulatory elements genome-wide [38]. We analyzed 90,857 transcription start sites (TSS) from csRNA-seq profiling of murine bone-marrow derived macrophages (BMDMs) activated by Kdo2-lipid A (KLA), as previously analyzed in Duttke et al. [38]. We searched for motifs within +/− 150 bp of the TSS, and scored them by their log_2_ fold change of csRNA-seq signal between KLA stimulation and control. MEIRLOP readily identified enrichment of motifs for the NF-κB and JUN/AP1 TF families in KLA-induced TSS (Fig. 4) [38]. These TFs are known to mediate KLA response [44]. In KLA-repressed TSS, MEIRLOP identified enrichment of motifs for PU.1/SPI1 (ETS), MITF (bHLH), and the MEF2 TF family (Fig. 4). These findings are consistent with previous literature indicating lipopolysaccharide (LPS) -induced downregulation of these TFs [45, 46].

### MEIRLOP identifies enriched TF binding motifs in DNase I hypersensitive sites

To further demonstrate the flexibility conferred by scored-based MEA, we used MEIRLOP to assign regulatory functions to TF motifs found enriched in DNase I Hypersensitive sites (DHS). Extensive chromatin profiling from ENCODE provides key information about the regulatory states at each DHS, although the levels of each chromatin modification are often correlated with the DNase hypersensitivity signal, which reflects the relative fraction of cells with open chromatin at that site in the population of cells [35, 36]. To identify DHS that are associated with specific epigenetic profiles independent of hypersensitivity levels, we computed composite scores across 235,220 DHS in K562 cells based on the log_2_ ratios of ChIP-seq coverage for the following scores: H3K27ac (active promoters and enhancers) over H3K27me3 (polycomb, repressive), reflecting active vs. repressed regulatory regions [7]; And H3K4me3 (promoters) over H3K4me1 (enhancers), reflecting DHS with promoter vs. enhancer characteristics [47]. Composite scores were derived from histone ChIP-seq coverage +/− 500 bp from the peak centers. To account for correlation of these scores with DNase hypersensitivity signal, we incorporated DNase-seq coverage +/− 75 bp from the peak centers as a covariate. We searched for motifs +/− 75 bp from the peak centers.

**Fig. 5:**
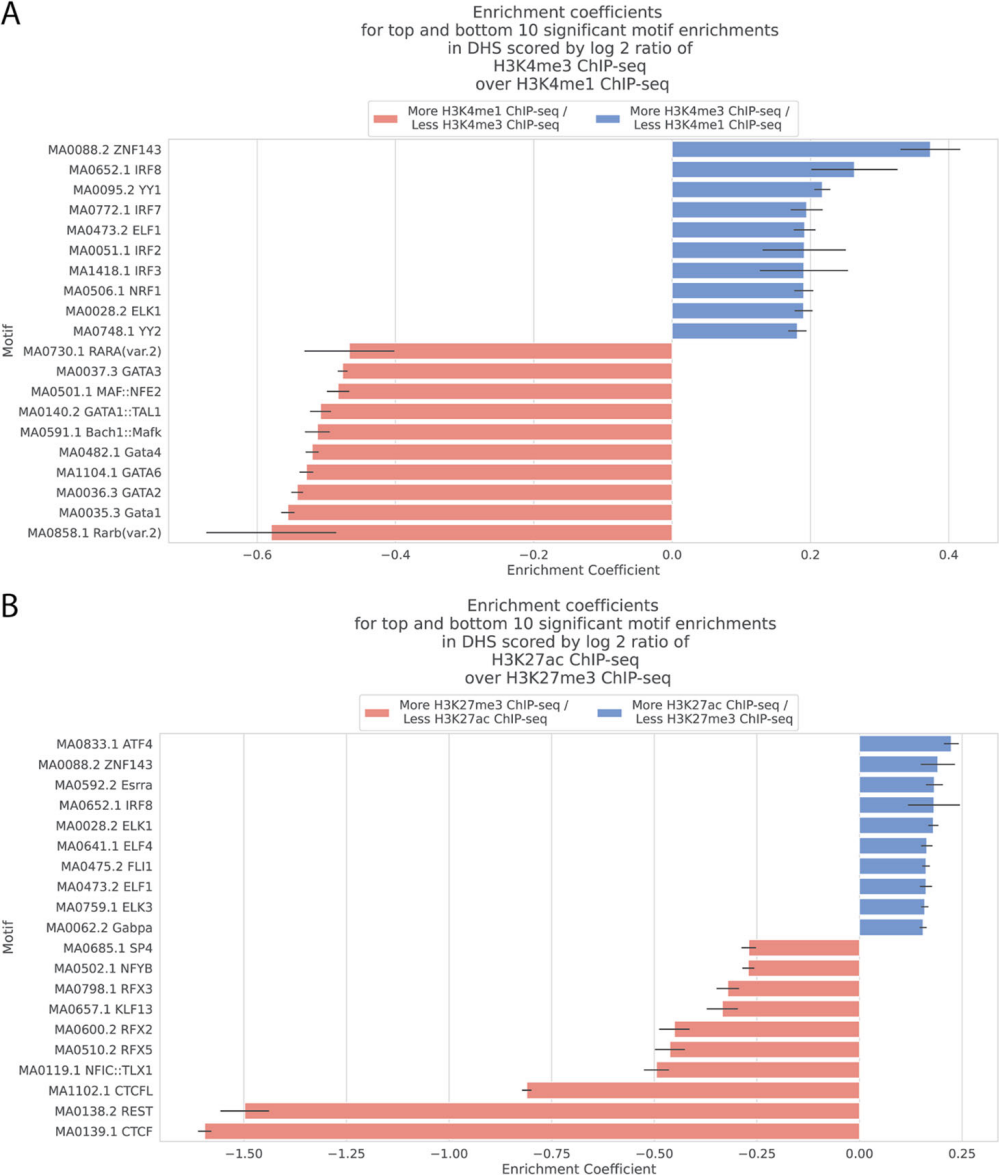
Logistic regression with covariates finds motifs for TFs associated with the ratios of different histone modifications over DHS. **a** Bar plot of enrichment coefficients for the top and bottom 10 most significantly enriched motifs associated with DHS scored by the log_2_ ratio of H3K4me3 over H3K4me1. Error bars represent the standard deviation of the enrichment coefficients. **b** Bar plot of enrichment coefficients for the top and bottom 10 most significantly enriched motifs associated with DHS scored by the log_2_ ratio of H3K27ac over H3K27me3. Error bars represent the standard deviation of the enrichment coefficients

MEIRLOP analysis of the H3K4me3/H3K4me1 composite ratio identified motifs for several TFs with known roles in promoters or enhancers. MEIRLOP found the motifs for ELF1, NRF1, and YY1 in the top 10 significant enrichments for more promoter-like DHS with a greater ratio of H3K4me3 over H3K4me1 (Fig. 5a). This finding has precedence in a larger study by Anderson et al., which also found motifs for these TFs significantly enriched in promoters compared to enhancers [48].The transcription factor ZNF143 also acts as a promoter-bound transcriptional activator, suggested to bind next to POL2 [49, 50].

In contrast, different sets of motifs were identified in enhancer-like DHS. Rye et al. previously found that NFE2 preferentially mapped to genomic regions marked with H3K4me1 compared to those marked with H3K4me3 [51]. DNA binding activity of GATA1 is correlated with H3K4me1, a histone marker for enhancers [48, 52]. However, the enhancer activity of DNA bound by GATA1 is associated with TAL1 co-binding [53, 54]. In keeping with these combined roles, MEIRLOP found a motif associated with binding of both TFs significantly enriched in DHS with greater ratios of H3K4me1 over H3K4me3 signal (enrichment coefficient = − 0.51, adjusted *p*-value < 0.01) (Fig. 5a). Significant enrichments for other GATA1-like motifs were also found enriched in DHS with greater H3K4me1 over H3K4me3 signal (Fig. 5a.).

Analysis of H3K27ac/H3K27me3 identifies the motifs of TFs that may contribute to highly active (high ratio) or repressed (low ratio) regions of chromatin. The high score for the ATF4 motif at active regions is consistent with the evidence that ATF4 recruits histone acetyltransferase [55]. At the other extreme, MEIRLOP found a binding motifs for CTCF and REST significantly enriched in more repressed DHS with higher levels of H3K27me3 over H3K27ac (enrichment coefficients ~ − 1.5, adjusted *p*-value < 0.01) (Fig. 5b). CTCF is an important factor in establishing 3D chromatin structure, while REST is a TF known to silence gene expression through chromatin remodeling [56, 57].

KLF13 (also known as BTEB3) represses transcription through interaction with histone deacetylases (HDACs) and competes with Sp1 for DNA-binding [58]. Notably, while MEIRLOP finds enrichment for a KLF13 binding motif associated with transcriptionally silenced chromatin (enrichment coefficient = − 0.33, adjusted p-value <1e-8) (Fig. 5b), it requires the additional DNase-seq signal covariate to do so: Running MEIRLOP with only dinucleotide-based covariates does not find a significant enrichment of this motif (enrichment coefficient − 4.8e-2, adjusted p-value = 0.18). This change in enrichment values is consistent with the moderate correlation of DNase-seq coverage and H3K27ac over H3K27me3 ratios across DHS (Spearman correlation coefficient = 0.51).

Overall, MEIRLOP found enrichments for TF motifs associated with key functional processes by analyzing DHS scored as a function of multiple sequencing assays, while also controlling for dinucleotide sequence bias and the quality of the DHS as a function of DNase-seq coverage. These enrichments are consistent with previous findings in the literature and known roles of the TFs binding to these motifs.

## Discussion

By incorporating covariates derived from low-level sequence bias (GC content, dinucleotide frequencies), MEIRLOP enables accurate score-based MEA that achieves accurate enrichment results. These results recapitulate the transcription factors involved in regulating transcriptional response to IFN-β stimulation. When applied to ENCODE TF ChIP-seq data, we find that our method performs just as well or better compared to multiple score-based MEA methods in recovering significant enrichment of the binding motif for the ChIPed TF. Combined with the faster execution of our implementation, this allows the application of our method in situations where researchers must analyze thousands of scored regulatory regions for motifs and TFs that can explain transcriptional regulation in differing conditions. Using a logistic regression model means that our method can also be extended to integrate covariates other than sequence composition, allowing researchers to control for variation in sequence length, batch effects, or systematic coverage biases found in control experiments.

Set-based MEA can remain applicable when the biological signal quantified across conditions for regulatory regions is bimodal, or when the signal can be thresholded into sizable sets of sequences. However, in scenarios where the list of significantly differentially regulated regions is small or the magnitude of regulation is weak, we demonstrated that score-based MEA can be sensitive enough to properly identify differential motif enrichment. But even where other score-based MEA methods may apply, we found that uneven distribution of GC content in the sequences analyzed obscured known biology from being recovered: If a researcher took results from methods that did not account for covariates at face value, then absent other evidence, they might conclude that homeobox transcription factors (with their GC-poor motifs) are more significant to transcriptional regulation of IFN-β response compared to interferon regulatory factors or STAT transcription factors.

We find that our method achieves accuracy similar to existing regression-based MEA methods on non-differential TF ChIP-seq data. TF ChIP-seq data quantifies the binding affinity of TFs to genomic regions, and so directly mirrors the assumption in AME’s regression-based methods that stronger binding motifs correlate with stronger binding. But even when applied to data that better reflects AME’s regression model, MEIRLOP remains competitive in both accuracy and runtime. Runtime improvements may result from: The use of a faster motif scanning algorithm provided by MOODS [24]; and the use of multithreading. However, the generalizability of the method may stem from its hybridization of set-based and score-based approaches: MEIRLOP treats motif presence as a Boolean variable and regulatory region activity as a continuous variable, rather than treating both as Boolean variables (as per a Fisher exact test) or both as continuous variables (as per regression). Since MEIRLOP takes arbitrary scores for regulatory region activity, it is able to analyze motif enrichments from novel sequencing methods, such as the recently developed csRNA-seq. However, reducing motif presence to a Boolean outcome variable can lead to loss of accuracy where motif matches may be inexact, e.g. where a modified TF does not bind as well to its canonical motif. Here, de novo motif finding methods can recover the true binding motif, then MEIRLOP can determine enrichment of this motif, as performed in Bloodgood et al. [59].

We demonstrated the flexibility of MEIRLOP in situations where the genomic regions quantified were scored using experimental assays that were different from those used to identify them: We found motif enrichments within DHS scored using composite measurements, as log_2_ ratios of histone ChIP-seq coverage across the DHS. MEIRLOP found multiple enriched motifs consistent with previous findings from the literature and with known roles of TFs corresponding to those motifs. This illustrates that MEIRLOP can determine motif enrichments along diverse scores of regulatory region activity, even where these scores may not have intuitive pre-defined thresholds due to their composite nature or relation to the genomic regions being analyzed. Furthermore, because MEIRLOP offers two-sided hypothesis testing, it enables researchers to investigate motifs enriched towards either extreme of such ratios in a single pass, instead of having to run a motif enrichment analysis tool twice to investigate both extremes. MEIRLOP’s ability to incorporate customized covariates also offers researchers further power to detect enrichments that are otherwise obscured by factors such as the quality of a DHS site, while also accounting for sequence bias. The combined flexibility from score-based motif enrichment and covariate control also serves MEIRLOP well when analyzing data from more recent sequencing strategies, including csRNA-seq. These examples indicate how MEIRLOP can serve researchers when performing motif-based analyses on data from newer techniques, where rule-of-thumb thresholds for set-based motif enrichment may not be fully established, enabling new findings.

## Conclusions

We have demonstrated that MEIRLOP can determine enrichment of sequence motifs where sequence bias or other covariates may confound other methods. To use MEIRLOP, researchers need only score genomic regions across a continuum of biological interest. These scores are flexible, and MEIRLOP’s two-sided enrichment can identify motifs enriched towards either extreme of log-ratios assembled from multiple sequencing experiments. Although MEIRLOP is confined to scoring the enrichment of a known motif library, future work will explore the de novo identification of motifs that maximize for enrichment based on MEIRLOP.

## Availability and requirements

**Project name:** MEIRLOP.

**Project home page:** https://github.com/npdeloss/meirlop

**Operating system(s):** Linux.

**Programming language:** Python 3.

**Other requirements:** Conda for installation and dependency management.

**License:** MIT License.

**Any restrictions to use by non-academics:** MIT License terms.

## Supplementary information

**Additional file 1: Figure S1.** Differential ChIP-seq of HCT116 cells before and after stimulation yields very few significantly differential peaks. **(A)** Volcano plot depicting Log_2_ fold change (Fold) and significance (negative of log *p*-value, neglogpval) of 20,087 peaks found using MACS2 and DiffBind for HCT116 cells with (*n* = 2) and without (*n* = 2) IFN-β stimulation. Peaks matching significantly differential criteria (FDR < 0.05, log 2 fold-change > 1.0) are highlighted in orange (*n* = 2).

**Additional file 2: Figure S2.** Logistic regression with covariates finds enrichment of IRF9 and STAT1::STAT2 binding motifs ahead of AT-rich homeobox binding motifs. **(A)** Top 10 significant enrichment results from our method, with and without covariates. Motifs are ordered as they appear in the HTML enrichment report output. **(B)** Top 10 significant enrichment results from other score-based MEA methods. Motifs are ordered as they appear in the HTML enrichment report output.

**Additional file 3: Table S3.** Table of ENCODE ChIP-seq datasets used. Contains detailed listings of ENCODE datasets used in results section “MEIRLOP achieves similar or better accuracy on TF ChIP-seq data”, including experiment accession IDs, download URLs, and the names of labs where the data were generated.

**Additional file 4: Table S4.** Table of ENCODE DNase-seq and histone ChIP-seq experiments used. Contains detailed listings of ENCODE datasets used in results section “MEIRLOP identifies enriched TF binding motifs in DNase I Hypersensitive Sites”, including experiment accession IDs, download URLs, and the names of labs where the data were generated.

## Data Availability

The ENCODE TF ChIP-seq datasets analysed in this study are available in the ENCODE repository, at https://www.encodeproject.org/ (doi: 10.1038/nature11247.). Detailed listings, including experiment accession IDs, download URLs, and the names of labs where the data were generated, are available in supplementary Table S3. Other ENCODE DNase-seq and histone ChIP-seq datasets in this study are also available in the ENCODE repository, at https://www.encodeproject.org/ (doi: 10.1038/nature11247.). Detailed listings, including experiment accession IDs, download URLs, and the names of labs where the data were generated, are available in supplementary Table S4. JASPAR 2018 CORE vertebrate non-redundant motif set file used in this study is available from the JASPAR 2018 archive download page, at http://jaspar2018.genereg.net/downloads/ [4]. The differential stimulation H3K27ac datasets used and/or analysed during the current study are available under GEO accession GSE147707. The code for MEIRLOP is available from its Github repository at https://github.com/npdeloss/meirlop, and can be installed through the Anaconda software distribution platform (for 64-bit Linux systems) with the command: “conda install -c bioconda -c conda-forge -c npdeloss meirlop” [20].

## Abbreviations

AME: Analysis of Motif Enrichment, software package
ChIP: Chromatin Immunoprecipitation
ChIPed TF: The transcription factor that is the immunoprecipitation target of a ChIP-seq assay; the ChIP-seq target transcription factor
CpG: Cytosine-phosphatase-Guanine
ENCODE: Encyclopedia of DNA Elements
FDR: False Discovery Rate
GC content: Guanine-Cytosine content
GSEA: Gene Set Enrichment Analysis, software package and method
H3K27ac: Histone H3 lysine 27 acetylation
IFN: Interferon
IRF: Interferon Regulatory Factor
ISGF3: Interferon-stimulated gene factor 3
KLA: Kdo2-lipid A
LPS: Lipopolysaccharide
MACS2: Model-based Analysis of ChIP-Seq, software package
MEA: Motif Enrichment Analysis
MEIRLOP: Motif Enrichment in Ranked Lists Of Peaks, software package
PWM: Position Weight Matrix
STAT: Signal Transducer and Activator of Transcription
TF: Transcription factor
TSS: Transcription Start Site
