# Supplementary figures and images for: MEIRLOP: improving score-based motif enrichment by incorporating sequence bias covariates
Source: BMC Bioinformatics. 2020 Sep 16;21:410. doi: 10.1186/s12859-020-03739-4 (PMC7493370; doi:10.1186/s12859-020-03739-4)

**Figure S1.**

**
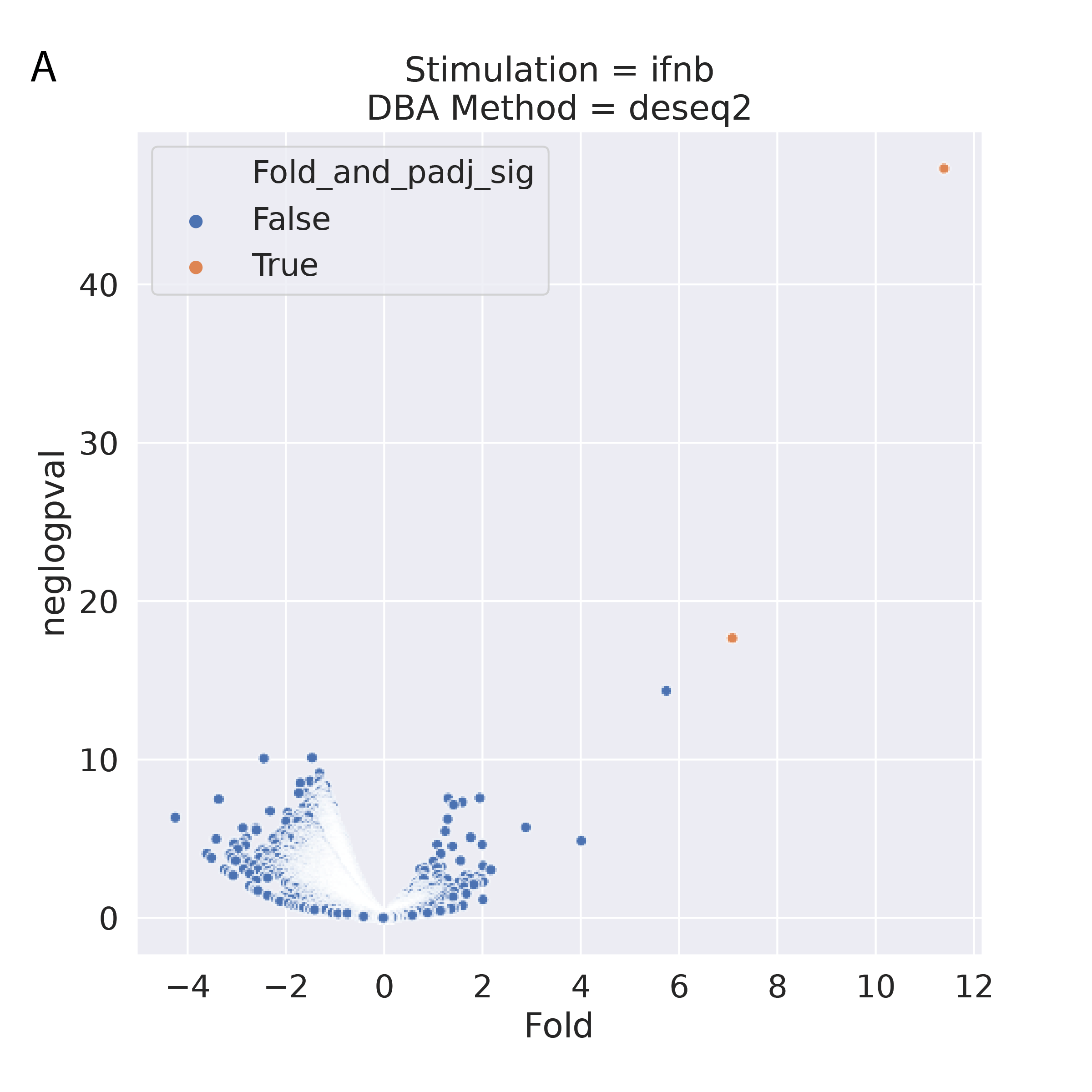
**

Supplement: Supplementary file 1 — Additional file 1: Figure S1. Differential ChIP-seq of HCT116 cells before and after stimulation yields very few significantly differential peaks. (A) Volcano plot depicting Log2 fold change (Fold) and significance (negative of log p-value, neglogpval) of 20,087 peaks found using MACS2 and DiffBind for HCT116 cells with (n = 2) and without (n = 2) IFN-β stimulation. Peaks matching significantly differential criteria (FDR < 0.05, log 2 fold-change > 1.0) are highlighted in orange (n = 2). [file 12859_2020_3739_MOESM1_ESM.docx]

**Figure S2.**


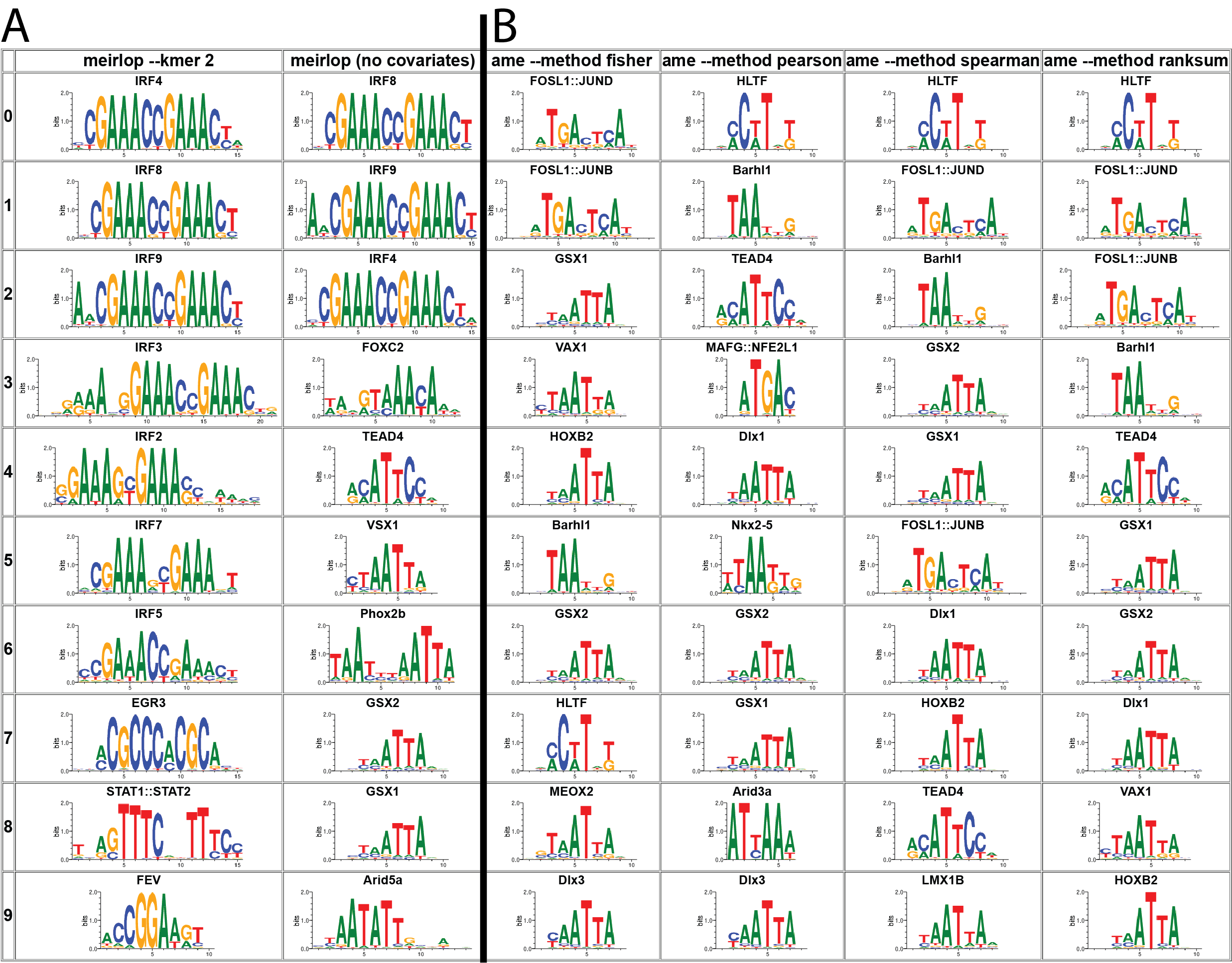

Supplement: Supplementary file 2 — Additional file 2: Figure S2. Logistic regression with covariates finds enrichment of IRF9 and STAT1::STAT2 binding motifs ahead of AT-rich homeobox binding motifs. (A) Top 10 significant enrichment results from our method, with and without covariates. Motifs are ordered as they appear in the HTML enrichment report output. (B) Top 10 significant enrichment results from other score-based MEA methods. Motifs are ordered as they appear in the HTML enrichment report output. [file 12859_2020_3739_MOESM2_ESM.docx]
